# Echocardiographic reference ranges in older children and adolescents in sub-Saharan Africa

**DOI:** 10.1016/j.ijcard.2017.06.109

**Published:** 2017-12-01

**Authors:** Edith D. Majonga, Andrea M. Rehman, Grace McHugh, Hilda A. Mujuru, Kusum Nathoo, Mohammad S. Patel, Shungu Munyati, Jon O. Odland, Katharina Kranzer, Juan P. Kaski, Rashida A. Ferrand

**Affiliations:** a London School of Hygiene and Tropical Medicine, London, United Kingdom; b Biomedical Research and Training Institute, Harare, Zimbabwe; c University of Zimbabwe, Harare, Zimbabwe; d MRI & Radiology Centre, Harare, Zimbabwe; e UiT, The Arctic University of Norway, Tromsø, Norway; f Department of Public Health, Faculty of Health Sciences, University of Pretoria, Pretoria, South Africa; g Centre for Inherited Cardiovascular Diseases, Great Ormond Street Hospital, London, United Kingdom; h Institute of Cardiovascular Science, University College London, United Kingdom

**Keywords:** Echocardiography, Reference ranges, Z-scores, Children, Africa

## Abstract

**Background:**

Echocardiographic reference ranges are important to identify abnormalities of cardiac dimensions. Reference ranges for children in sub-Saharan Africa have not been established. The aim of this study was to establish echocardiographic z-score references for Black children in sub-Saharan Africa.

**Methods:**

282 healthy subjects aged 6–16 years (143 [51%] males) with no known history of cardiac disease were enrolled in the study in Harare, Zimbabwe between 2014 and 2016. Standard M-mode echocardiography was performed and nine cardiac chamber dimensions were obtained. Two non-linear statistical models (gamma weighted model and cubic polynomial model) were tested on the data and the best fitting model was used to calculate z-scores of these cardiac chamber measures. The reference ranges are presented on scatter plots against BSA.

**Results:**

Normative data for the following cardiac measures were obtained and z-scores calculated: right ventricular diameter at end diastole (RVEDD); left ventricular diameter at end diastole (LVEDD) and systole (LVESD); interventricular septal wall thickness at end diastole (IVSd) and systole (IVSs); left ventricular posterior wall thickness at end diastole (LVPWd) and systole (LVPWs); left atrium diameter at end systole (LA) and tricuspid annular plane systolic excursion (TAPSE). Girls had higher values for BMI and heart rate than boys (*p* = 0.048 and *p* = 0.001, respectively). Mean interventricular septal and left ventricular posterior walls thickness was higher than published normal values in predominantly Caucasian populations.

**Conclusion:**

These are the first echocardiographic reference ranges for children from sub Saharan Africa and will allow accurate assessment of cardiac dimensions in clinical practice.

## 1 Introduction

Transthoracic echocardiography enables non-invasive assessment of cardiac size and function and is an essential tool for cardiac evaluation in children and adults. As cardiac chamber dimensions can change with somatic growth in childhood and adolescence, it is important to normalise echocardiographic measurements to body size. Several echocardiographic references have been published for children and adolescents from various regions including Europe, Asia and North America [1], [2], [3], [4], [5]. However, no echocardiography references have been established for children and adolescents in sub Saharan Africa, and echocardiographic studies from this region have utilised published references for normative data mostly derived from predominantly white populations [4], [6]. Racial differences in cardiac dimensions have been reported from previously published nomograms with Black race children found to have significantly larger cardiac dimensions than White race children [7]. In addition, the standards of growth for a population may be influenced by environmental, social and economic factors of that region. This necessitates development of regional echocardiographic references. The aim of this study was to establish echocardiographic z-score references in Black African children.

## 2 Methods

The study was conducted at the Harare Children's Hospital, Zimbabwe, between August 2014 and December 2016. Children aged between 6 and 16 years who were HIV-uninfected were invited to participate in the study. Participants were recruited from seven primary care clinics in Harare that were offering HIV-testing to all attendees regardless of the reason for presentation as part of a project evaluating HIV testing services. The study was also advertised at the hospital to the general public. Those who tested HIV-negative were given a flyer with information about the study and asked for consent to be contacted for possible inclusion in the study. Participants were included if they had no known congenital and/or acquired cardiac disease, no cardiac symptoms, a normal electrocardiogram (ECG) and no evidence of structural or functional heart disease on echocardiography. The age, height and weight were obtained and body surface area (BSA) was calculated using Dubois and Dubois method [8].

Ethical approval was obtained from Harare Central Hospital Ethics Committee, Medical Research Council of Zimbabwe, London School of Hygiene and Tropical Medicine Ethics Committee and Biomedical Research and Training Institute Institutional Review Board. Written informed consent from guardians and assent from participants were obtained prior to enrolment in all cases.

### 2.1 Echocardiographic examination

Echocardiography was performed using a Mindray DC N6 multipurpose ultrasound machine (Mindray, Shenzhen, China) by a trained and experienced paediatric echocardiographer (EM). 2D guided M-mode echocardiography was performed on all children using a standard protocol, according to published guidelines [9]. No sedation was required prior to the examination. Participants were scanned in the left lateral or supine position to obtain an optimum image quality. Images were acquired using a transducer with frequencies ranging from 3.5 MHz to 7.0 MHz and simultaneous 3-lead ECG monitoring and were saved in DICOM format for subsequent off-line analysis.

The following cardiac measures were obtained over three cardiac cycles: right ventricular diameter at end diastole (RVEDD); left ventricular diameter at end diastole (LVEDD) and end systole (LVESD); interventricular septal wall diameter at end diastole (IVSd) and end systole (IVSs); left ventricular posterior wall thickness at end diastole (LVPWd) and end systole (LVPWs); left atrium diameter at end systole (LA); and tricuspid annular plane systolic excursion (TAPSE). End diastole was defined as the start of the QRS-wave on the ECG tracing, or preferably described as the frame in the cardiac cycle in which LV dimension is largest and systole as the frame prior to mitral valve opening in which LV dimension is smallest, as previously described [10]. Measurements were performed in the parasternal long axis (PLAX) or parasternal short axis (PSAX) views using the leading edge to leading edge method [11]. TAPSE was measured in apical 4-chamber view. Measurements were only made on technically adequate images, thus not all measurements were obtained in every patient (Table 1). Repeated measurements were performed on 20 randomly selected echocardiograms by the same rater (EM) and a further 28 (10%) of all the echocardiograms were randomly selected and re-measured by an independent rater (MSP).

**Table 1:** Clinical and echocardiographic characteristics of participants.

Variable	Overall *N* = 282	Males *N* = 143	Females *N* = 139	*P*-value
Age (y)	10.7 (3.0)	10.7 (3.1)	10.7 (2.9)	0.922
*6–9 years*	*107 (38)*⁎	*55 (38)*⁎	*52 (37)*⁎	
*10–13 years*	*107 (38)*⁎	*51 (36)*⁎	*56 (40)*⁎	
*14–16 years*	*68 (24)*⁎	*37 (26)*⁎	*31 (22)*⁎	
Height (cm)	140.3 (16.2)	140.4 (17.5)	140.2 (14.8)	0.920
Weight (kg)	35.6 (13.2)	34.3 (12)	37 (14.3)	0.008
BSA (m^2^)	1.18 (0.3)	1.16 (0.3)	1.19 (0.3)	0.421
BMI (kg/m^2^)	17.4 (3.1)	16.8 (2.0)	18.1 (3.9)	**0.001**⁎⁎
Systolic BP (mmHg)	112.7 (12.9)	112 (13.0)	113 (12.9)	0.571
Diastolic BP (mmHg)	74 (9.6)	73 (9.0)	74.7 (9.7)	0.205
Heart rate (bpm)	81.7 (13.0)	80.2 (12.0)	83.3 (13.7)	**0.048**⁎⁎
RV diastole (mm)	14.9 (2.0)	14.9 (2.0)	14.9 (2.0)	0.969
LV diastole (mm)	39.2 (4.1)	39.7 (4.0)	38.7 (4.1)	**0.036**⁎⁎
LV systole (mm)	26.8 (3.1)	27 (3.1)	26.5 (3.2)	0.170
IVS diastole (mm)	7.5 (1.3)	7.6 (1.3)	7.5 (1.3)	0.303
IVS systole (mm)	9.9 (1.7)	10 (1.8)	9.8 (1.6)	0.473
LVPW diastole (mm)	7.3 (1.1)	7.4 (1.1)	7.2 (1.1)	0.253
LVPW systole (mm)	9.8 (1.7)	9.8 (1.7)	9.7 (2.9)	0.511
Left atrium (mm)	25.6 (3.4)	25.7 (3.3)	25.5 (3.5)	0.579
***TAPSE (mm)	20.7 (2.3)	20.7 (2.1)	20.8 (2.4)	0.873

*Results are****mean* *±*** ***SD****.*

*BSA, body surface area; BMI, body mass index; BP, blood pressure; RV, right ventricle; LV, left ventricle; IVS, interventricular septum; LVPW, left ventricular posterior wall; TAPSE, tricuspid annular plane systolic excursion.*

*⁎* *N(%)****.***

*⁎⁎* *Significant at p* *≤* *0.05.*

*⁎⁎⁎* *N* *=* *237.*

### 2.2 Statistical analysis

Data were analyzed using STATA version 12 software (StataCorp, Texas, USA). Continuous variables are presented as mean ± standard deviation (SD) for normally-distributed data or median and interquartile range (IQR) for non-normally-distributed data. To compute the z-scores, two regression models were tested on the data to optimise goodness of fit for the various cardiac measures and the selected independent variable, BSA. The two models were a gamma function weighted model [y = αx^β^ × e^− λx^] proposed by Nevill et al. [12] and a cubic polynomial model [y = ax^3^ + bx^2^ + cx + d] used by Petterson et al. [5] The final best model was selected based on several characteristics: first, by visual inspection of the goodness of fit of data plotted on a graph; secondly, the association between the residual and/or z-score plots against the BSA were assessed and no significant association should be observed if there is an adequate fit; thirdly, residual values were assessed for normal distribution; fourthly, the tails of distributions were assessed to ensure that individuals falling outside of the reference ranges (i.e. individuals with z-scores > + 2 or <− 2) were < 2.28% [13] to ensure that no bias was introduced and finally the model with the smallest Akaike information criterion (AIC) value was selected. Intra-rater and inter-rater reliability was calculated using Intra-class correlation coefficient (ICC). A value of *p* < 0.05 was considered statistically significant.

## 3 Results

A total of 282 children were enrolled in the study, of whom 143 (51%) were male. All participants were in sinus rhythm. The baseline demographic and clinical characteristics are shown in Table 1, stratified by gender. Girls had higher values for BMI and heart rate than boys (*p* = 0.048 and *p* = 0.001, respectively) whereas boys had higher LVEDD dimensions (*p* = 0.036).

**Fig. 1:**
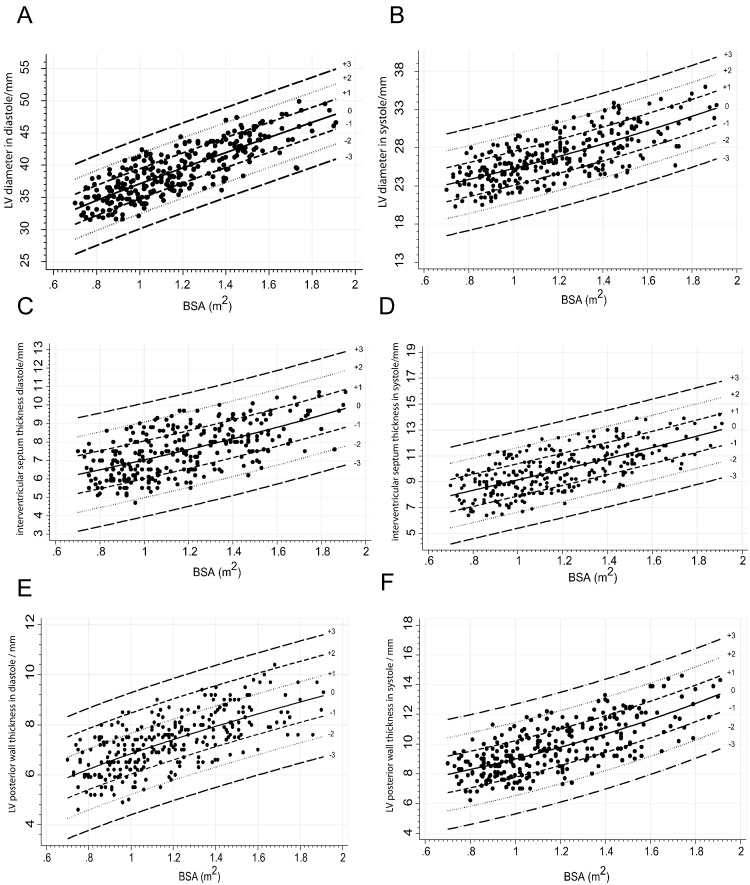
*Scatter plots for left ventricular dimensions against BSA****.*** *BSA, body surface area; LV, left ventricle* *The scatter plots show left ventricular related chamber dimensions.****A,****left ventricular diameter end diastole;****B,****left ventricular diameter end systole;****C***, *interventricular septum thickness end diastole;****D***, *interventricular septum thickness end systole;****E***, *left ventricle posterior wall thickness end diastole;****F***, *left ventricle posterior wall thickness end systole.*

**Fig. 2:**
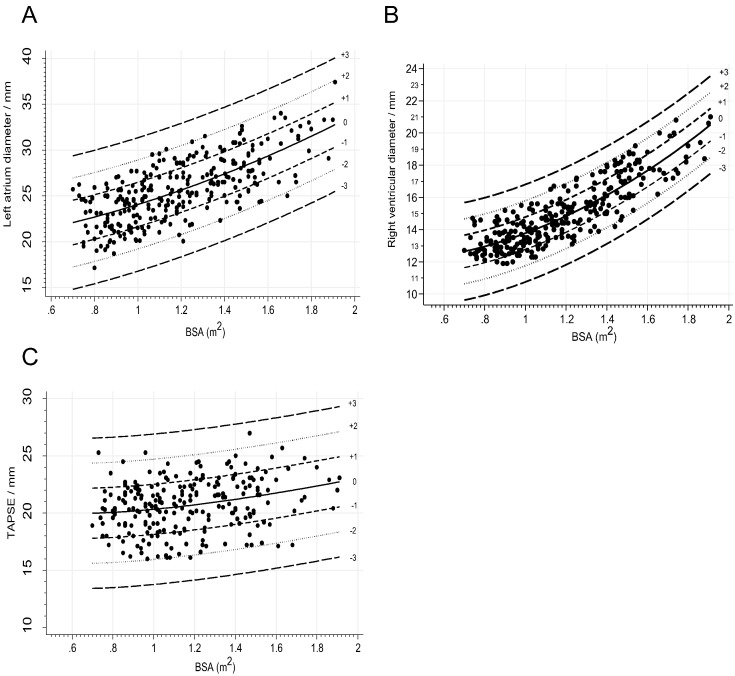
*Scatter plots for left atrium, right ventricle and TAPSE against BSA****.*** *BSA, body surface area; TAPSE, tricuspid annular plane systolic excursion;* *The scatter plots are showing A, left atrium diameter end systole; B, right ventricular diameter end diastole; C, TAPSE (N* *=* *232).*

Both the gamma function weighted and the cubic polynomial models had comparable visual goodness-of-fit of the data on plotted graphs and no significant association was observed between residuals and BSA. However, the gamma weighted model was selected because of the higher R^2^ values and smaller AIC values compared to the cubic polynomial model. In addition, the cubic polynomial did not have enough observations in the tail of the distribution. The regression analysis coefficients of the gamma weighted model are presented in Table 2. These include alpha, beta, lambda, root mean squared error (RMSE) and the r-squared (R^2^) values. The scatter plots of the cardiac measures against BSA are shown in Fig. 1, Fig. 2. The various lines in each graph represent the regression equations for z-sores ranging from − 3 to + 3, with 0 as the predicted or mean value. The z-scores for the children can either be approximated using these graphs or may be calculated by substituting the coefficients in Table 2 directly into the gamma equation. The z-scores are calculated using the standard formula: (X − μ) / σ where X is the observed value, μ is the predicted mean and σ is the standard deviation. For example, a z-score for a 14-year-old male with a BSA of 1.3m^2^ and measured LA of 30 mm, is calculated as follows:

**Table 2:** Coefficients of the gamma weighted model for the echocardiographic measures.

Measurement	Alpha	Beta	Lambda	AIC	RMSE	R^2^
RV diastole	7.086	− 0.320	− 0.665	808.1	1.009	0.996
LV diastole	32.223	0.198	− 0.141	1281.1	2.333	0.997
LV systole	18.623	− 0.010	− 0.306	1253.8	2.223	0.993
IVS diastole	5.119	0.071	− 0.317	817.7	1.026	0.982
IVS systole	7.064	0.183	− 0.259	927.1	1.246	0.985
LVPW diastole	6.508	0.381	− 0.050	687.6	0.8145	0.988
LVPW systole	5.555	− 0.063	− 0.481	921.4	1.233	0.985
Left atrium	15.774	− 0.116	− 0.422	1303.7	2.428	0.991
TAPSE	16.287	− 0.139	− 0.222	1047.7	2.193	0.989

*AIC, Akaike information criterion; RMSE, root mean squared error; R*^*2*^*, r-squared; RV, right ventricle; LV, left ventricle; IVS, interventricular septum; LVPW, left ventricular posterior wall; TAPSE, tricuspid annular plane systolic excursion.*

The mean LA is predicted using the coefficients in Table 2 in the equation. Y, is the value to be predicted and X is the BSA.

$$y={\mathrm{\alpha X}}^{\beta }\times e^{-\mathrm{\lambda x}}$$

Therefore, the mean predicted LA is: Y = 15.774 × 1.3^(− 0.116)^ × e^−(− 0.422 × 1.3)^ = 26.481. The z-score is calculated as: (30–26.481) / 2.428 = 1.449.

Thus, the boy has an approximate z-score of + 1.45.

Inter-rater and intra-rater reliability was good for all cardiac measures, with an ICC ≥ 0.88 and ≥ 0.87, respectively.

## 4 Discussion

This study is the first to establish normal reference ranges for M-mode echocardiography measures among older children and adolescents in sub Saharan Africa. The results have important clinical implications, as they will allow more accurate assessment of cardiac dimensions in clinical practice and in future research studies.

Throughout childhood, cardiac size changes dramatically. This presents a challenge in the interpretation of echocardiographic measures in the paediatric population [5]. Unlike in adults, where reference ranges have been presented and applied as a single “normal range”, this approach is imprecise in children as body size needs to be considered [14]. *Z*-scores quantify the degree of normality or abnormality by the number of standard deviations above or below a size-specific population mean of a particular measure [14]. A normal range of z-scores for the cardiac measures is defined as + 2 to − 2 with 0 as the mean. To date, no consensus exists as to which growth parameter is most appropriate in standardizing cardiac measures for children [15]. Different parameters have been used in the published references, including height [16], weight [3], BSA [2], [4], lean body mass [17], [18] and age [19], [20], [21]. Roge et al. showed that height, weight and BSA were strongly correlated, resulting in similar regression equations using either of the parameters [22]. We chose to express our results in relation to BSA as this is the most widely used parameter in clinical practice. Age alone is an unreliable standardizing parameter because at any age of a child, height, weight and BSA are variable [23].

Several authors have published M-mode references in non-African children standardised to BSA [5], [7], [24], [25]. Our references have a higher mean for interventricular septum and posterior wall thickness compared to the published references. Left ventricular and atrial diameters are comparable to Kampmann et al. [24] and Huwez et al. [25]. Right ventricular diameter results for our study are higher than those reported by Kampmann et al., and Huwez et al., while being lower than those of Bonatto et al. [7] and Petterson et al. [5]. Our TAPSE values are comparable to Nunez-Gil et al. [26] among Spanish children and lower than Uysal et al. [27] among Turkish children. These variations may be explained by differences in race and environment. Only Caucasians were included in Kampmann's cohort, while other studies did not mention the race of the children studied [5], [25]. Bonatto et al. studied Black and White children and found that Black children had overall larger cardiac dimensions than White children, consistent with the higher measurements reported in the present study and highlighting the importance of having specific reference ranges for sub Saharan populations. To date, no previous studies have focused specifically on this population.

Several authors have reported gender differences in their cohorts, mainly due to variations of growth of boys and girls, particularly at puberty [6], [28], [29]. In our study, no significant gender differences were observed on the cardiac measures, except for LVEDD. The difference in LVEDD between boys and girls was small and a possible explanation for this difference could be differences in growth rates between boys and girls.

The strength of this study is the relatively large sample size and the fact that reference ranges have been prospectively derived from a homogenous group of Black African children from the same racial group. Our population was not completely unselected and there may be some selection bias. Nevertheless, only healthy children with no known heart disease were included. We have established an important set of normal cardiac measurements for clinical practice. Lack of ethnic variation may be viewed as a limitation. However, as Black African children have not had representative references in the past, it allows comparison of these findings with data from other racial groups. We were not able to measure all parameters in all the children, particularly TAPSE (45 were missing) due to technically inadequate apical 4-chamber views. Our study is limited in that not all echocardiograms were interpreted by an independent reader; however, 10% of randomly selected echocardiograms were re-measured by the independent reader and the inter-rater reliability was good, suggesting that the results are valid. Furthermore, we followed a standard protocol to acquire images and perform measurements and, in clinical practice, echocardiograms are interpreted mostly by a single person. No two-dimensional (2D) measures, chamber volumes and functional parameters were measured and establishment of these normative values will be the focus of future studies in sub-Saharan populations. However, although M-mode is known to have reduced spatial resolution compared to 2D echocardiography [11], it has higher temporal resolution and remains the most commonly used method in clinical practice to assess cardiac chamber size and/or function due to its reproducibility and ease of performance. Given that our population is limited to only Zimbabwean children, the findings in this study may not be applicable to other African populations.

To our knowledge, this is the first study to establish echocardiography reference ranges in Black African children in sub Saharan Africa. These reference standards can be used for both clinical practice and research studies. Addition of our references to existing literature will allow a more precise assessment of cardiac dimensions in children.

## Funding

This work was supported by the Nina Ireland Program for Lung Health and the Wellcome Trust (095878/Z/11/Z). Salary support for AMR was provided by the UK Medical Research Council through a grant to the LSHTM Tropical Epidemiology Group; grant code MR/K012126/1.

## Conflict of interest

No conflict of interest.
